# Bariatric Surgery Should Be Offered to Active-Duty Military Personnel: a Retrospective Study of the Canadian Armed Forces’ Experience

**DOI:** 10.1007/s11695-023-06455-z

**Published:** 2023-01-28

**Authors:** Olivier Mailloux, Nicolas Tassé, André Tchernof, Mélanie Nadeau, Philip Dawe, Andrew Beckett, Laurent Biertho

**Affiliations:** 1 Royal Canadian Medical Services, Canadian Armed Forces, Ottawa, Ontario, Canada; 2 Department of Surgery, Laval University, Québec City, Québec G1V 0A6 Canada; 3 CISSS Côte-Nord-Hôpital Le Royer, 635 Blvd Jolliet, Baie-Comeau, Québec G5C1P1 Canada; 4 General Surgery Residency Program, Laval University, Québec City, Québec G1V 0A6 Canada; 5 Bariatric and Metabolic Surgery Research Chair – Laval University, Québec City, Québec G1V 0A6 Canada; 6 School of Nutrition, Laval University, Québec City, Québec G1V 0A6 Canada

**Keywords:** Bariatric surgery, Military, Obesity

## Abstract

**Purpose:**

Like most Western armies, obesity affects Canadian Armed Forces (CAF) personnel. Bariatric surgery is an effective treatment for obesity. However, this is not yet accepted for active-duty soldiers in most countries. The CAF have approved bariatric surgery since 2005. Our aim is to assess weight loss, resolution of obesity-related comorbidities, and impacts of bariatric surgery on military careers.

**Materials and Methods:**

We retrospectively reviewed the perioperative data, long-term bariatric results, and military outcomes of 108 CAF active-duty military personnel who underwent bariatric surgery in Canada over a 61-month period.

**Results:**

The cohort was predominantly male (66.7%) with a mean preoperative body mass index (BMI) of 43.6 ± 5.8 kg/m^2^. Roux-Y gastric bypass was performed in 59 patients, sleeve gastrectomy in 29, and gastric banding in 20. All the surgeries were performed laparoscopically. The total body weight loss at the last follow-up visit was 22.5 ± 11.0%. Remission or improvement of hypertension was observed in 91.2%, diabetes in 85.7%, gastroesophageal reflux disorder (GERD) in 43.6%, sleep apnea in 43.1%, and dyslipidemia in 42.9%. One patient (0.9%) was medically released due to postoperative complications. Fifteen patients (13.9%) were deployed postoperatively. The combined deployable and possibly deployable statuses increased from 35.4% preoperatively to 47.9% postoperatively.

**Conclusion:**

This is the largest series of bariatric surgeries performed in active-duty military personnel. Bariatric surgery is effective and safe and improves deployability without impairing military careers. These results are relevant to the military of many industrialized countries. Bariatric surgery should be considered for all active-duty military personnel who meet surgical criteria for the treatment of obesity.

**Graphical Abstract:**

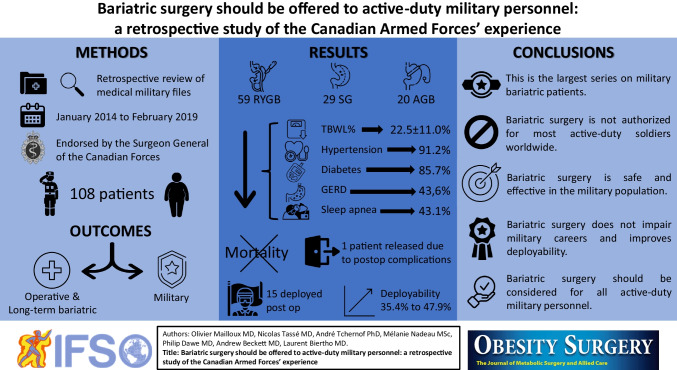

## Introduction

Obesity has become a global epidemic. Over 650 million people meet or exceed the body mass index (BMI) threshold of 30 kg/m^2^ [1]. In 2017, 35.8% of Canada’s population was reported to be overweight and 26.5% were obese [2]. As a strain on the Canadian economy, obesity is estimated to cost 1.27 to 11.08 billion dollars a year, with diabetes and hypertension costing 10,023 and 2341 dollars a year, respectively [3–5].

This obesity epidemic has reached the military population as well and is not limited to the civilian population. Most Western militaries report an obesity rate ranging from 10 to 20%, and annual obesity-related costs are reaching billions of dollars [6–12]. The Canadian Armed Forces (CAF) personnel are no exception and show a trend similar to that of the Canadian civilian population. As much as 49.0% of regular force personnel are classified as overweight and 25.0% as obese [13].

Bariatric surgery is recognized as the most effective and durable treatment for severe obesity and its comorbidities in the civilian population [14]. In the military population, bariatric surgery has been poorly studied. A literature search performed by the investigators has identified a single study published on active-duty soldiers who underwent bariatric procedures [15, 16]. Although bariatric surgery appears to be an effective treatment option for active-duty soldiers and would logically not compromise their future ability to deploy or meet the universality of service requirements, there is insufficient data to reach a firm conclusion. This lack of data is partially explained by the fact that many occidental armed forces do not allow bariatric procedures for their active-duty personnel [17]. Indeed, bariatric procedures are often considered conditions for potential release [18]. However, the CAF approved bariatric surgery for its active personnel more than a decade ago [19, 20].

Herein, we report the outcomes of a CAF cohort of active-duty military personnel who underwent bariatric surgery. Our hypothesis is that bariatric surgery is at least as effective in military population as in the literature on civilian populations and that it favorably impacts military careers. Our primary objective was to assess weight loss and the resolution of obesity-related comorbidities such as sleep apnea, hypertension, GERD, dyslipidemia, and diabetes. Secondly, we evaluated the impact of surgery on military careers through deployability and personnel retention.

## Materials and Methods

This study was approved by the Canadian Surgeon General Health Research Program and the Quebec Heart and Lung Institute Research Ethics Committee protocol number 2021–3587,22024. We retrospectively reviewed all files of CAF active-duty military personnel who underwent bariatric surgery between January 2014 and February 2019. All patients met the indications for bariatric surgery [14, 21]. Clinical and perioperative data were obtained from the electronic medical records and the Canadian Force Health Information System (CFHIS). The CAF Blue Cross Registry was consulted for financial and surgical data. To evaluate obesity-related comorbidities, the American Society for Metabolic and Bariatric Surgery outcome reporting standards were used. We employed subjective assessment for sleep apnea and GERD. It was also used to record complications [22].

We also reviewed military data such as rank, postoperative deployment, and medical categories using the CFHIS. Canadian medical categories are a numeric profile composed of six factors assigned to each individual CAF member. It summarizes key information about a member’s employability, deployability, and medical fitness [23]. We recorded the preoperative and postoperative geographic (G) and occupational (O) factors. These are the only factors that may be affected by bariatric surgery. The geographic factor is defined as being in relation to the environment that a member can or cannot tolerate. This definition has evolved based on the required proximity to medical care. This includes the requirement for scheduled medical care as well as an assessment of the risk of recurrence or exacerbation of the medical condition and the level of medical care that would be required. The occupational factor is defined as the physical and mental activity and the stress associated with employment within a specific military occupational structure identification code (MOSID); although often difficult to describe and measure in an objective and reproducible manner, it is an important aspect in the grading of the occupational factor. The demands on the member may vary with the MOSID as well as with the geographical location. To assess deployability for members who did not deploy postoperatively, we created four deployability statuses based on a combination of G and O factors [(1) deployable (≤ G3/O2), (2) Possibly deployable (≤ G4/O3), (3) non-deployable (≥ G5/ ≥ O4), and (4) non-assessable (e.g., on other medical restrictions)]. We used the Medical Risk Matrix to define the first three categories [24]. The non-assessable category included patients on temporary categories, which is defined as a non-permanent G and O factor, which are reassessed usually every 3–9 months.

Continuous variables were reported as mean ± standard deviation. Normal distribution was assessed with the Shapiro–Wilk statistical test using a significance threshold of *p* < 0.05 for non-normality. We performed Student’s *t*-tests to compare normally distributed variables between groups. Non-normally distributed variables were analyzed using the non-parametric Wilcoxon test. Preoperative and postoperative measurements were compared in a paired fashion using paired *t*-tests. Categorical data were reported as frequencies and comparisons were performed using Fisher’s exact test. All statistical analyses were performed using the JMP statistical software (SAS Institute Inc., Cary, NC, USA).

## Results

The study group comprised 108 patients. The cohort was predominantly male (66.7%). The mean age of the patients was 42 years, and the mean preoperative BMI was 43.6 kg/m^2^. A minority of patients were officers (*N* = 9, 8.3%). Characteristics are presented in Table 1.

**Table 1:** Preoperative demographics (*n* = 108)

Gender	
• Male	72
• Female	36
Age (years)	42.2 ± 4.8
Preoperative weight (kg)	131.2 ± 23.7
Preoperative body mass index (kg/m^2^)	43.6 ± 5.8
Smoking	26 (24.1%)
Abdominal surgery history	39 (36.4%)
Officers	9
Comorbidities	
• Musculoskeletal disorders	94 (87.0%)
• Sleep apnea	68 (63.0%)
• Hypertension	47 (43.9%)
• GERD	39 (36.1%)
• Dyslipidemia	35 (32.4%)
• Diabetes	31 (28.7%)

Data presented as mean ± standard deviation or number of patients (percentage of sample)

The operative data are summarized in Table 2. Three types of surgeries were performed. Roux-Y gastric bypass (RYGB) was the most frequent operation (*n* = 59), followed by sleeve gastrectomy (SG) (*n* = 29) and adjustable gastric band (AGB) (*n* = 20). The last AGB was conducted in 2016. All the surgeries were performed laparoscopically. Surgeries were performed by seven (7) surgeons from seven (7) clinics (five private and two public) in three provinces of Canada (Ontario, Québec, and British Columbia). The patients underwent surgery mainly in private clinics (106) and the average cost was 18,062$CAD. Revision surgery was performed in five patients. Three patients had their AGB removed and transformed into RYBG (2) or SG (1). Two SG were converted to a RYGB. No revision surgery was performed for weight gain. There was no mortality. Seven (6.5%) patients had early major complications within 30 days of surgery, and all underwent RYBG. Two patients required laparoscopic drainage for anastomotic leaks. We found that nine patients (8.7%) had late major complications, most of which were related to AGB. Minor early and late complications occurred in 11 (10.2%) and 22 (20.4%) patients, respectively.

**Table 2:** Operative characteristics (*n* = 108)

Surgical procedures	
• Roux-Y gastric bypass	59 (54.6%)
• Sleeve gastrectomy	29 (26.9%)
• Adjustable gastric band	20 (18.5%)
Laparoscopic approach	108 (100%)
Conversion	0
Revision procedure	5
Complications	
Mortality	0
Early major complications (≤ 30 days)	7 (6.5%)
• *Upper GI bleeding*	3
• *Anastomotic leaks*	2
• *Deep vein thrombosis/pulmonary embolism*	2
Early minor complications (≤ 30 days)	11 (10.2%)
• *Superficial site infection*	7
• *Nausea*	3
Late major complications (> 30 days) (*n* = 103)	9 (8.7%)
• *Gastric band complications*	4
• *Perforated anastomotic ulcer*	1
• *Gastro-gastric fistula*	1
• *Incisional hernia*	1
• *Undiagnosed eating disorder*	1
• *Intractable vomiting requiring surgical conversion*	1
Late minor complications (> 30 days) (*n* = 104)	22 (20.4%)

Data presented as number of patients (percentage of sample)

Postoperative characteristics are listed in Table 3. Data was available for 106 patients. Follow-up ranged from 4 to 60 months, with a mean last follow-up of 31.3 ± 18.5 months. All bariatric procedures significantly reduced body weight (101.3 ± 22.5 kg, *p* < 0.0001) and BMI (33.7 ± 6.3 kg/m^2^, *p* < 0.0001). Hypertension remission or improvement was observed in 91.2%, diabetes in 85.7%, sleep apnea in 43.1%, and dyslipidemia in 42.9% of cases. In 43.6% of patients, GERD subjectively resolved or improved, while 6 (8.7%) developed GERD. Among them were three RYGB, one SG, and two AGB, of which one was converted to RYGB. Medication use was significantly reduced for all comorbidities.

**Table 3:** Postoperative outcomes

Weight loss (*n* = 106)		
• Last follow-up (month)		31.3 ± 18.5
• Weight at last follow-up (kg)		101.3 ± 22.5
• Weight loss at last follow-up (kg)		29.6 ± 15.8
• Body mass index (kg/m^2^)		33.7 ± 6.3
• Total body weight loss (TBWL) (%)		22.5 ± 11.0
• Excess weight loss (EWL) (%)*		49.3 ± 25.0
Co-morbidities		
• Sleep apnea (*n* = 65)		
◦ Improvement		28 (43.1%)
◦ Unchanged		37 (56.1%)
• Hypertension (*n* = 41)		
◦ Complete remission		4 (9.8%)
◦ Partial remission		15 (36.5%)
◦ Improvement		18 (43.9%)
◦ Unchanged		4 (9.8%)
• GERD (*n* = 39)		
◦ Complete resolution		16 (41.0%)
◦ Improvement		1 (2.6%)
◦ Unchanged		22 (56.4%)
◦ New onset (*n* = 69)		6 (8.7%)
• Dyslipidemia (*n* = 21)		
◦ Remission		3 (14.3%)
◦ Improvement		6 (28.6%)
◦ Unchanged		12 (57.1%)
• Diabetes (*n* = 28)		
◦ Complete remission		15 (53.6%)
◦ Partial remission		3 (10.7%)
◦ Improved		6 (21.4%)
◦ Unchanged		3 (10.7%)
◦ Recurrence		1 (3.6%)
Medication use	*Preoperative*	*Postoperative*
• Hypertension (*n* = 47)	1.4 ± 0.8	0.5 ± 0.8 (*p* < 0.0001)
• GERD (*n* = 39)	0.9 ± 0.4	0.6 ± 0.5 (*p* < 0.0001)
• Dyslipidemia (*n* = 35)	0.8 ± 0.4	0.3 ± 0.5 (*p* < 0.0001)
• Diabetes (*n* = 31)	1.4 ± 1.0	0.4 ± 0.8 (*p* < 0.0001)

Data presented as mean ± standard deviation or number of patients (percentage of sample)

^*^With BMI 23 kg/m^2^ as reference

Data specific to military personnel are summarized in Table 4. For medical categories, the geographic factors were improved or unchanged in 33.3% and 48.0% of the patients, respectively. Occupational factors improved or remained unchanged in 24.5% and 57.8% of the patients, respectively. Fifty-one patients (47.2%) were released from service on medical grounds. Of these, 16 patients were released before their surgery. The main causes for medical releases were post-traumatic stress disorder (PTSD), musculoskeletal disorders, and mental health issues. Only one patient was directly released because of postoperative complications, which was an anastomotic leak complicated by a gastro-gastric fistula.

**Table 4:** Military outcomes

Medical categories (*n* = 102)	
Geographic (G) factor modification	
• Improved	34 (33.3%)
• Unchanged	49 (48.0%)
• Deteriorated	19 (18.6%)
Occupational (O) factor modification	
• Improved	25 (24.5%)
• Unchanged	59 (57.8%)
• Deteriorated	18 (17.6%)
Military release	53 (49.1%)
• Retirement/voluntary	2
• Medical	51
◦ Post-traumatic stress disorder	18
◦ Musculoskeletal disorder	17
◦ Mental health issues	9
◦ Cardiovascular disease	3
◦ Bariatric postoperative complications	1
◦ Complicated diabetes	1
◦ Vertigo	1
◦ Unavailable	1
• Released before surgery	16
Deployability	
• Postoperative deployment (*n* = 108)	15 (13.9%)
Deployability by medical categories	
• Preoperative (*n* = 107)	
◦ Deployable	30 (28.0%)
◦ Possibly deployable	8 (7.4%)
◦ Not deployable	24 (22.4%)
◦ Not assessable	45 (42.1%)
• Postoperative (*n* = 102)	
◦ Deployable	36 (35.2%)
◦ Possibly deployable	13 (12.7%)
◦ Not deployable	46 (45.1%)
◦ Not assessable	7 (6.9%)

Data presented as mean ± standard derivation or number of patients (percentage of ample)

Fifteen patients (13.9%) were deployed postoperatively. Deployed personnel had a significantly greater weight loss at their last follow-up (37.5 ± 15.6 kg, *p* < 0.05) and a tendency for higher TBWL (27.3 ± 9.1%, *p* = 0.06) than those who were not deployed (28.1 ± 15.6 kg and 21.5 ± 11.1%). Based on medical categories (G/O factors), the combined deployable and possibly deployable status increased from 35.4% before surgery to 47.9% postoperatively. The non-assessable status decreased from 42.1 to 6.9%. Compared to deployable and possibly deployable, undeployable patients did not differ in major complications, both early and late (1 vs. 3, NS; 7 vs. 1, *p* = 0.0615), as well as in minor complications, both early and late (4 vs. 5, NS; 12 vs. 6, NS).

## Discussion

To the best of our knowledge, this is the largest series of bariatric surgeries performed on active-duty military personnel, and the second one reported to date. Brounts et al. reported in 2009 on a series of 27 active military patients who underwent open RYGB [16]. The mean age was 34.2 with a mean perioperative BMI of 40.6 kg/m^2^. This went down to 25.6 kg/m^2^ postoperatively. A total of 24 patients maintained or achieved a deployable status after surgery. Only one patient could not remain in active duty because of chronic complications related to bariatric surgery. From a surgical technique standpoint, our series is more contemporary with RYGB and SG being the predominant techniques used, and all surgeries being performed laparoscopically, as reported in the surgical literature [25].

Our military population is quite different from that of civilian Canadian bariatric patients (apart from age and smoking habits) [26]. One major difference was the low prevalence (33.3%) of female patients in our cohort. However, considering that the female military represents only 16% of all CAF personnel [27], the proportion of women is still high, much like that in the civilian population. In addition, comorbidities are more frequent and in different proportions compared to civilian patients, with an increased rate of musculoskeletal disorders (87%). Although these injuries may be service-related, they are often underreported and combined with off-duty traumas; thus, the relationship between military employment and obesity remains unclear. Officers were underrepresented in this series from a military perspective. They composed only 8.3% of the sample, whereas officers represented 24.8% of the CAF [3]. The exact explanation for this difference is unclear, but could be related to different socioeconomic patterns, as reported in the bariatric literature [28].

When compared to the bariatric literature, surgeries in military personnel appear to produce comparable results [29]. As expected, a significant reduction in body weight and BMI was observed. Comorbidities were also diminished in a similar fashion, especially for diabetes and hypertension. Aside from the obvious medical advantages, military organizations also benefit financially. By removing 18 diabetic and 19 hypertensive patients from their medication, the CAF saves are estimated to be 248,303$CAD annually, theoretically recovering the surgery costs in less than 3 years [4, 5].

Moreover, our data suggest that it is safe to perform bariatric surgery on active-duty military personnel, with no mortality and complication rates within what is reported in the literature, both in type and frequency. A more direct comparison of civilian and military data is required and intended by the investigators. Interestingly, all early major and late complications occurred in RYGB patients. As military personnel can be deployed in remote areas with scarce health resources, the risk of complications such as internal hernia, ulcers, or small bowel obstruction should be considered.

In addition to the direct healthcare benefits, our data also present an advantage from a military standpoint. Medical categories were either improved or unchanged for most patients following surgery. The proportion of deployable and possibly deployable statuses increased by 12.5% following surgery. Although the nondeployable status increased by 22.7%, the nonassessable status showed the largest change with a 35.2% reduction. These results may indicate that surgical treatment of obesity and correction of its comorbidities may help military clinicians better evaluate medical categories, resulting in a net increase in potentially deployable personnel. More importantly, fifteen (13.9%) patients were deployed without adverse events associated with their surgery. Only one nondeployed patient was released for medical reasons related to a surgical complication. This is a major finding that may encourage military leadership to allow bariatric surgery for active-duty personnel.

This study also had some limitations. The retrospective design implies bias and limitations. Follow-up data were obtained through military clinics instead of bariatric clinics and were more focused on bariatric data. Although CAF has a national electronic medical record system, the data available from medical files vary greatly. Follow-up was affected by this and by the high number of patients who were released from military work and were lost to follow-up. As a result, patients were no longer available for follow-up through military medical charts, and we were no longer able to identify them. Medical records also did not register members’ specific military employments. With military professionalization, new and more sedentary professions are now part of modern armies. This variation in different physical demands could not be assessed here. We also have three different surgeries with outcomes known to be different, but we lacked the power to compare them. Regarding the high number of medical releases during the study period, most were related to PTSD and mental health issues and not to bariatric surgery. This demonstrates the reality of the challenges faced by military personnel and health services. Sixteen patients were released before surgery, but still underwent the procedure while in the military. This is secondary to the CAF policy on personnel retention [30]. In certain trades where there is a shortage of personnel, medically released members may remain in uniform for an additional 3 years. Twenty-two (43.1%) releases were related to either musculoskeletal disorders, cardiovascular diseases, or diabetes. Although we were unable to determine if an earlier surgery would have prevented these releases, this may be a plausible hypothesis. Another limitation is the deployability status inferred by medical categories (G and O factors). Although a member may be healthy enough to deploy, many other factors, either geopolitical, unit-, or work-related, may prevent deployment. Therefore, we used medical categories as proxies to mitigate these external factors. The deployability status based on medical categories was developed by the authors to correlate with the CAF Medical Risk Matrix [20]. This tool is designed to predict the likelihood of future reoccurrence of a medical condition and its operational consequences in the theater, and itself has its limitations.

An additional limitation was the impossibility to access members’ FORCE Evaluation results, the CAF annual fitness test [31]. These data are not charted in medical records and many members were medically exempted preoperatively. To assess physical fitness, we used medical categories as a surrogate, and it may not be as precise or quantifiable as the annual fitness test. Further studies should include more objective physical fitness data.

Furthermore, the surgeries were mainly performed in private clinics until 2018. They were also performed in only three of the 10 Canadian provinces. This may have impacted the uniformity of surgical care and bariatric follow-up. Although civilian Canadian health services are provincial public systems, private care may have granted military personnel better access to bariatric surgery. Indeed, a small number of civilian-eligible patients have access to bariatric surgery in Canada through its public system, and there are great discrepancies among provinces [32]. The CAF has recently decided to no longer authorize surgery in the private sector [19].

## Conclusion

To the best of our knowledge, this is the largest series of bariatric surgeries performed in active-duty military personnel to date. For members of the Canadian Armed Forces, bariatric surgery is both effective and safe. In addition, the data show that it is not detrimental to military careers and may improve deployability. If offered earlier, this could help prevent irreversible medical conditions and avoid members from being medically released. The retention of trained personnel and experience is critical to armed forces. We suggest that these results may be relevant to the military of many industrialized countries. Bariatric surgery should be considered for all active military personnel who meet the standard surgical indications for the management of obesity.

## References

[1] World Health Organization Consultation on Obesity. Obesity: preventing and managing the global epidemic, Report of a WHO Consultation [Internet]. Geneva, Switzerland: World Health Organization; 1999. [Cited 2019 February 5]. Available from: https://apps.who.int/iris/handle/10665/42330. Accessed 5 Feb 2019.

[2] Table 13-10-0373-01. Overweight and obesity based on measured body mass index, by age group and sex [Internet]. Statistics Canada; 2020 [Cited 2022 March 30]. Available from: 10.25318/1310037301-eng.

[3] Davis JA (2020). Comparison of comorbidity treatment and costs associated with bariatric surgery among adults with obesity in Canada. JAMA Netw Open..

[4] Rosella LC (2016). Impact of diabetes on healthcare costs in a population-based cohort: a cost analysis. Diabet Med..

[5] Weaver CG (2015). Healthcare costs attributable to hypertension: Canadian population-based cohort study. Hypertension..

[6] Quertier D, Goudard Y, Goin G (2022). Overweight and Obesity in the French Army. Mil Med..

[7] Military Health System and Defense Health Agency. Department of Defense Health of the Force 2020 [Internet]. Falls Church, VA: Department of Defense; 2020. [Cited 2021 March 7] Available from: https://www.health.mil/Reference-Center/Reports?query=health%20of%20force&isDateRange=0&broadVector=000&newsVector=00000000&refVector=000000000000100&refSrc=1.

[8] Sanderson PW (2014). Prevalence and socio-demographic correlates of obesity in the British Army. Ann Hum Biol..

[9] Samito S (2013). Physical fitness in dependence on cardiovascular risk factors - an assessment of 20- to 30-year-old adults. Gesundheitswesen.

[10] Fajfrova J (2016). Prevalence of overweight and obesity in professional soldiers of the Czech Army over an 11-year period. Vojnosanit Pregl..

[11] Gaździńska A (2022). Assessment of risk factors for development of overweight and obesity among soldiers of polish armed forces participating in the National Health Program 2016-2020. Int J Environ Res Public Health..

[12] Tompkins E. Obesity in the United States and effects on military recruiting [Internet]. Washington, DC: Congressional Research Service; 2020 December 22. Report No.: IF11708 – Version:3. [Cited 2021 March 7]. Available from: https://crsreports.congress.gov/product/details?prodcode=IF11708.

[13] Thériault FL, Gabler K, Naicker K (2016). Health and lifestyle information canadian forces personnel 2013/2014 – regular force report [Internet].

[14] American Society for Metabolic and Bariatric Surgery (ASMBS) [Internet]. Newberry, FL: Bariatric Surgery Guidelines and Recommendations, June 2012. [cited 2019 February 6]. Available from: https://asmbs.org/app/uploads/2014/06/Bariatric-Guidelines-and-Recommendations-1.pdf.

[15] Mailloux O, Dhillon P. Bariatric surgery in active-duty military personnel, a literature review. In: Poster presented at: The Canadian Institute for Military and Veteran Health Research (CIMVHR) 10th Annual Conference. Gatineau, Québec; 2019.

[16] Brounts LR, Lesperance K, Lehmann R (2009). Resectional gastric bypass outcomes in active duty soldiers: a retrospective review. Surg Obes Relat Dis..

[17] TRICARE Program. Surgery for Morbid Obesity [Internet]. Feder Regist. 2011;76(30):8294–8298. [Cited 2019 March 30]. Available from: https://www.govinfo.gov/content/pkg/FR-2011-02-14/pdf/2011-3207.pdf. 21348347

[18] Department of Defense Health Affairs. Memorandum for Assistant Secretary of the Army (M&RA), Assistant Secretary of the Navy (M&RA), Assistant Secretary of the Air Force (M&RA), Director, Joint Staff [Internet]. Washington, DC, United States of America: The Assistant Secretary of Defense; 2007 May 11. Report No.: Health Affairs Policy 07-006. [cited 2019 March 30]. Available from: https://tricare.mil/CoveredServices/IsItCovered/BariatricSurgery.

[19] Canadian Armed Forces Spectrum of Care – Comprehensive medical care services / Bariatric Surgery [Internet]. Government of Canada. [cited 2019 March 30]. Available from: https://www.canada.ca/en/department-national-defence/services/benefits-military/pay-pension-benefits/benefits/medical-dental/medical-care-services.html#bariatric-surgery.

[20] Director of Medical Policy. Guidance for bariatric surgery referrals. Ottawa, ON, Canada: Canadian Forces Health Services Groupe; 2017 September 19. Report No.: Communiqué 2017–2001.

[21] Wharton S, Lau DCW, Vallis M (2020). Obesity in adults: a clinical practice guideline. CMAJ..

[22] Brethauer SA (2015). Standardized outcomes reporting in metabolic and bariatric surgery. Surg Obes Realt Dis..

[23] A-MD-154-000/FP-000. Canadian Armed Forces Medical Standards (CFP 154) [Internet]. Governement of Canada; 2017. [cited 2021 November 3]. Available from: https://www.canada.ca/en/department-national-defence/corporate/policies-standards/medical-standards-military-occupations.html.

[24] A-MD-154-000/FP-000. Annex F - Medical Risk Matrix. Incorporation of a risk management process in the establishment of medical employment limitations [Internet]. Government of Canada; 2018. [cited 2021 November 3]. Available from: https://www.canada.ca/en/department-national-defence/corporate/policies-standards/medical-standards-military-occupations/medical-risk-matrix.html.

[25] Alalwan AA, Friedman J, Park H (2021). US national trends in bariatric surgery: A decade of study. Surgery.

[26] Padwal RS, Chang HJ, Sharma AM (2012). Characteristics of the population eligible for and receiving publicly funded bariatric surgery in Canada. Int J Equity Health..

[27] Statistics of women in the Canadian Armed Forces [Internet]. Government of Canada. [Updated 2022 May; cited 2021 December 31]. Available from: https://www.canada.ca/en/department-national-defence/services/women-in-the-forces/statistics.html.

[28] Hernandez B, Voll S, Lewis NA (2021). Comparisons of disease cluster patterns, prevalence and health factors in the USA, Canada, England, and Ireland. BMC Public Health.

[29] Dan AG (2016). Metabolic and bariatric surgery. Surgical Clinics of North America.

[30] DAOD 5023-1, Minimum operational standards related to universality of service [Internet]. Governement of Canada; 2018. [cited 2021 December 31]. Available from: https://www.canada.ca/en/department-national-defence/corporate/policies-standards/defence-administrative-orders-directives/5000-series/5023/5023-1-minimum-operational-standards-related-to-universality-of-service.html#amosi.

[31] Evaluation FORCE. Fitness for Operational Requirements of Canadian Armed Forces Employment [Internet]. Canadian Forces Morale & Welfare Services. 2022; [cited 2022 September 2022]. Available from: https://cfmws.ca/sport-fitness-rec/fitness-testing/cmtfe-force-evaluation/force-evaluation.

[32] Sharma AM (2016). Inequalities in access to bariatric surgery in Canada. CMAJ.

